# Surface-Enhanced Raman Scattering Spectroscopy for Label-Free Analysis of *P. aeruginosa* Quorum Sensing

**DOI:** 10.3389/fcimb.2018.00143

**Published:** 2018-05-11

**Authors:** Gustavo Bodelón, Verónica Montes-García, Jorge Pérez-Juste, Isabel Pastoriza-Santos

**Affiliations:** Departamento de Química Física y Centro Singular de Investigaciones Biomédicas (CINBIO), Universidad de Vigo, Vigo, Spain

**Keywords:** quorum sensing, bacteria, imaging, metabolites, *Pseudomonas aeruginosa*, SERS, raman scattering

## Abstract

Bacterial quorum sensing systems regulate the production of an ample variety of bioactive extracellular compounds that are involved in interspecies microbial interactions and in the interplay between the microbes and their hosts. The development of new approaches for enabling chemical detection of such cellular activities is important in order to gain new insight into their function and biological significance. In recent years, surface-enhanced Raman scattering (SERS) spectroscopy has emerged as an ultrasensitive analytical tool employing rationally designed plasmonic nanostructured substrates. This review highlights recent advances of SERS spectroscopy for label-free detection and imaging of quorum sensing-regulated processes in the human opportunistic pathogen *Pseudomonas aeruginosa*. We also briefly describe the challenges and limitations of the technique and conclude with a summary of future prospects for the field.

## Introduction

During their growth, bacteria secrete a large repertoire of chemical compounds that can function in the environment as signaling molecules, cues, virulence factors and agents of microbial warfare (Phelan et al., 2011; Ratcliff and Denison, 2011; Davies and Ryan, 2012; Davies, 2013). These bioactive compounds are involved in competitive strategies, and other community behaviors, such as biofilm formation and syntrophy, and they are believed to play a major role on the survival of the producing organisms in the natural environment (O'Brien and Wright, 2011; Stubbendieck and Straight, 2016; van der Meij et al., 2017). Besides their influence in the ecology of microbial communities, bacterial extracellular compounds have a direct impact in human health and disease, as they have been associated with infection, inflammation, cancer, as well as neurological disorders, and their expression has been correlated to changes in the composition of the human microbiota (Peters et al., 2012; Garg et al., 2017). Many of these biomolecules display a remarkable range of drug-like bioactivities, and thereby they have been used as a source of antibiotics, chemotherapeutic drugs, immune suppressants and crop protection agents for biomedicine and agricultural applications (Newman and Cragg, 2007; Harvey et al., 2015). However, despite the myriad of compounds with pharmaceutical interest identified so far, their true biological role and the ecological significance remain poorly characterized (Davies, 2013). In this respect, recent studies have shown that at sub-inhibitory concentrations, molecules released by bacterial cells bearing antibiotic capacity can modulate gene expression, acting in the natural environment as molecules for signaling, cueing and chemical manipulation (Bernier and Surette, 2013). Indeed, the general term “antibiotic,” commonly used to describe antibacterial drugs, overlooks its suspected range of biological activities. In this context, it has been proposed that whether a microbial compound acts as an antimicrobial agent, signal, cue, or coercion, depends on the fitness consequences of the interaction (Diggle et al., 2007).

The production of an ample array of extracellular bioactive compounds is often regulated under quorum sensing (QS) systems (Antunes et al., 2010; Popat et al., 2015). In general, the QS systems of Gram-negative bacteria include an enzyme that synthesizes the signaling molecule and a transcription factor that binds to the signal modulating the expression of QS regulons, including upregulation of the synthase. This “autoinduction” positive feedback loop promotes synchronous gene expression in the population (Papenfort and Bassler, 2016). It is firmly established that QS plays global regulatory roles in bacterial metabolism, virulence, and contributes to the modulation of bacterial antibiotic tolerance and host defense mechanisms. The early observation that QS mutants of clinically-relevant pathogens have greatly reduced virulence has spurred an explosion of research aimed at targeting QS as a potential therapeutic avenue to treat bacterial infections (LaSarre and Federle, 2013; Whiteley et al., 2017).

Gram-negative *P. aeruginosa* is a ubiquitous and highly versatile opportunistic human pathogen that can cause acute and severe biofilm-related chronic infections, which can readily develop multi-drug resistance leading to high morbidity and mortality rates, especially in immunocompromised and cystic fibrosis (CF) patients. Significantly, the number of multi-drug and pan-drug resistant strains of this pathogen is increasing worldwide, complicating therapeutics (Poole, 2011). The ubiquitous presence of this organism, as well as its prevalence and persistence in clinical settings is attributed to its extraordinary capability of adaptation and survival, in which QS has a central regulatory role (Moradali et al., 2017). The QS network of *P. aeruginosa* is comprised by at least four QS systems that are highly interconnected and function in a hierarchical way (Lee and Zhang, 2014; Papenfort and Bassler, 2016). The sophisticated QS regulatory mechanisms present in *P. aeruginosa* are mainly involved in signaling, virulence determinant production, motility, biofilm development, antibiotic resistance mechanisms, as well as the adjustment of metabolic pathways and physiological processes in response to environmental cues and stresses, endowing this organism with the capacity to colonize different ecological niches and thrive in multispecies communities.

Several lines of evidence indicate that QS is implicated in the virulence of *P. aeruginosa* in human infections. Most isolates of this microorganism preserve functional QS systems, and QS signals are detected in biofluids of infected patients, which correlates with active QS expression during infection (Castillo-Juárez et al., 2015). In the context of polymicrobial infections, it is recognized the potential impact of interspecies interactions in disease severity and antibiotic efficacy (Peters et al., 2012). Studies investigating interactions between *P. aerigunosa* and *Staphylococcus aureus*, frequently isolated from the lungs of CF patients and chronic wounds, have shown that QS-regulated extracellular compounds produced by these microorganisms strongly influence the interaction between the coexisting bacterial species leading to phenotypes with decreased susceptibility to antibiotic treatment (i.e., persister cells, small colony variants) and worse disease outcomes (Hotterbeekx et al., 2017). A recent study has shown that alginate overproduction by *P. aeruginosa* during the conversion to mucoid phenotypes promotes coexistence with *S. aureus* in the CF lung (Limoli et al., 2017). *P. aeruginosa* and strains of *Burkholderia cepacia* can also co-exist in CF airways. It has been shown that *P. aeruginosa* can activate the QS system of *B. cepacia* (Riedel et al., 2001), and *P. aeruginosa*-derived rhamnolipids can modulate biological responses in *Burkholderia* spp. at low concentrations (Bernier et al., 2017). Similarly, it has been reported QS-based interactions between *P. aeruginosa* and *Candida albicans* in polymicrobial communities of these typical pneumonia pathogens (Fourie et al., 2016). The nature of the different bacterial processes controlled by QS in infections is currently an active area of research. It is believed that a clearer understanding of how QS-regulated extracellular compounds are used by *P. aeruginosa* to interact with other organisms and influence their local environment, as well as the conditions under which these molecules are expressed, could yield valuable information to assist the rational development of novel therapeutic drugs and improved therapeutics to treat microbial infections. In this framework, the ability to detect these chemical compounds with high sensitivity, and to non-invasively visualize their spatiotemporal distributions in live multispecies microbial communities is fundamental to provide new insights into their function, as well as the spatial dependencies required for chemical crosstalk.

Surface-enhanced Raman scattering (SERS) spectroscopy is an analytical tool that combines the molecular specific information provided by Raman scattering with the signal-enhancing power of plasmonic nanostructures. Through SERS it is possible to harness chemical information of biomolecules without the need of any external labeling (i.e., label-free), as well as non-invasive analysis of biological samples and imaging of cells (Cialla-May et al., 2017; Kahraman et al., 2017; Laing et al., 2017). Based on its high sensitivity and spectral resolution, SERS has been applied successfully to trace analysis, reaching single-molecule detection level under favorable conditions (Nie, 1997). Owing to significant key advantages, SERS has emerged in microbiology research for chemical profiling of microbial cells (Liu et al., 2017; Lorenz et al., 2017), detection and identification of bacteria at different taxonomic levels (Pahlow et al., 2015; Rebrošová et al., 2017), single cell analysis (Kuku et al., 2017), or *in vivo* diagnostics and multimodal imaging (Henry et al., 2016; Cialla-May et al., 2017; Krafft et al., 2017).

In this review we aim to highlight recent applications of SERS spectroscopy for label-free detection and imaging of *P. aeruginosa* extracellular compounds in the context of QS communication. Since in this specific topic there are still few examples in the literature, our objective is to introduce this technology to interested readers, as well as to pinpoint current challenges and limitations of SERS as an analytical tool for the detection of microbial extracellular biomolecules, as well as other classes of SERS-active cellular compounds.

## Raman scattering and SERS spectroscopy

**Figure 1:**
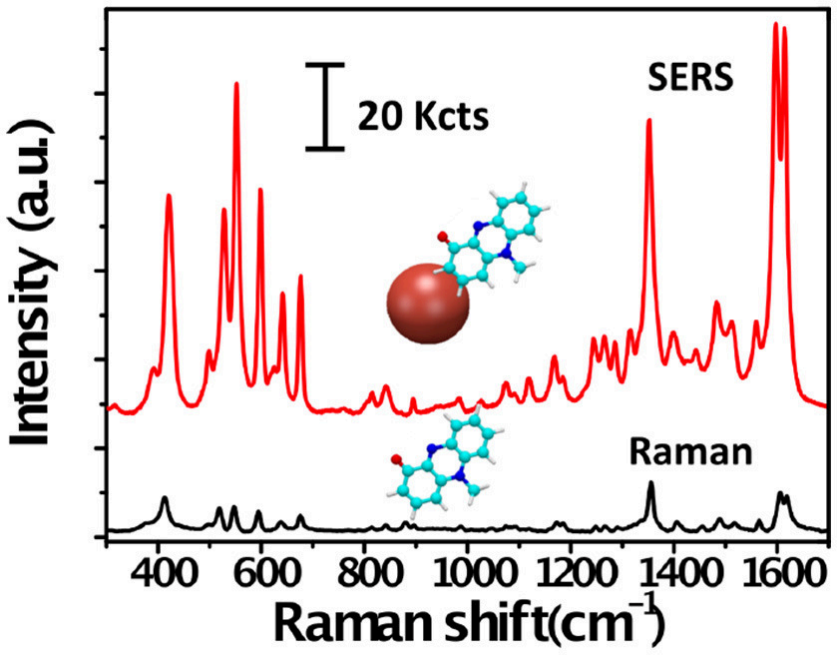
Raman and SERS spectra of pyocyanin. Raman (black) and SERS (red) spectra of pyocyanin bound to a plasmonic gold nanoparticle (red sphere). The peaks in the Raman spectrum correspond to vibrational modes of the molecule. Note the difference in intensity between the Raman and SERS signals of pyocyanin.

Raman scattering may be defined as the inelastic scattering of photons by molecular bond vibrations. The detection of scattered photons from a molecule yields a spectrum of Raman peaks, each of which is characteristic of a specific molecular bond, thereby allowing molecular identification on the basis of specific vibrational fingerprints (Figure 1). As compared with fluorescence and infrared spectroscopy, the higher spectral resolution and narrower bandwidths that characterize the Raman spectra facilitate the simultaneous detection of different analytes in multiplex analysis. In addition, the linear dependence of the Raman signal intensity on the analyte concentration offers the possibility for quantitative analysis (Schlücker, 2014). However, the Raman scattering signal is very weak, as only a very small fraction of the incident photons are scattered inelastically (about 1 out of 10 millions), whereby only high concentration of molecules can be detected, seriously limiting the application of this technique. SERS is a surface phenomenon that can amplify the inherently weak Raman scattering signal of molecules adsorbed, or in close vicinity, on a plasmonic metal nanoparticle when it is excited with an appropriate laser wavelength (Schlücker, 2014). Under such conditions single molecule detection levels can be reached, while retaining the structural information provided by Raman scattering (Figure 1). In SERS, average enhancement factors range between 10^4^ and 10^8^, and even values about 10^11^ can be achieved in some cases (Prochazka, 2016). This has rendered SERS spectroscopy a powerful analytical technique for ultrasensitive chemical or biochemical analysis.

**Figure 2:**
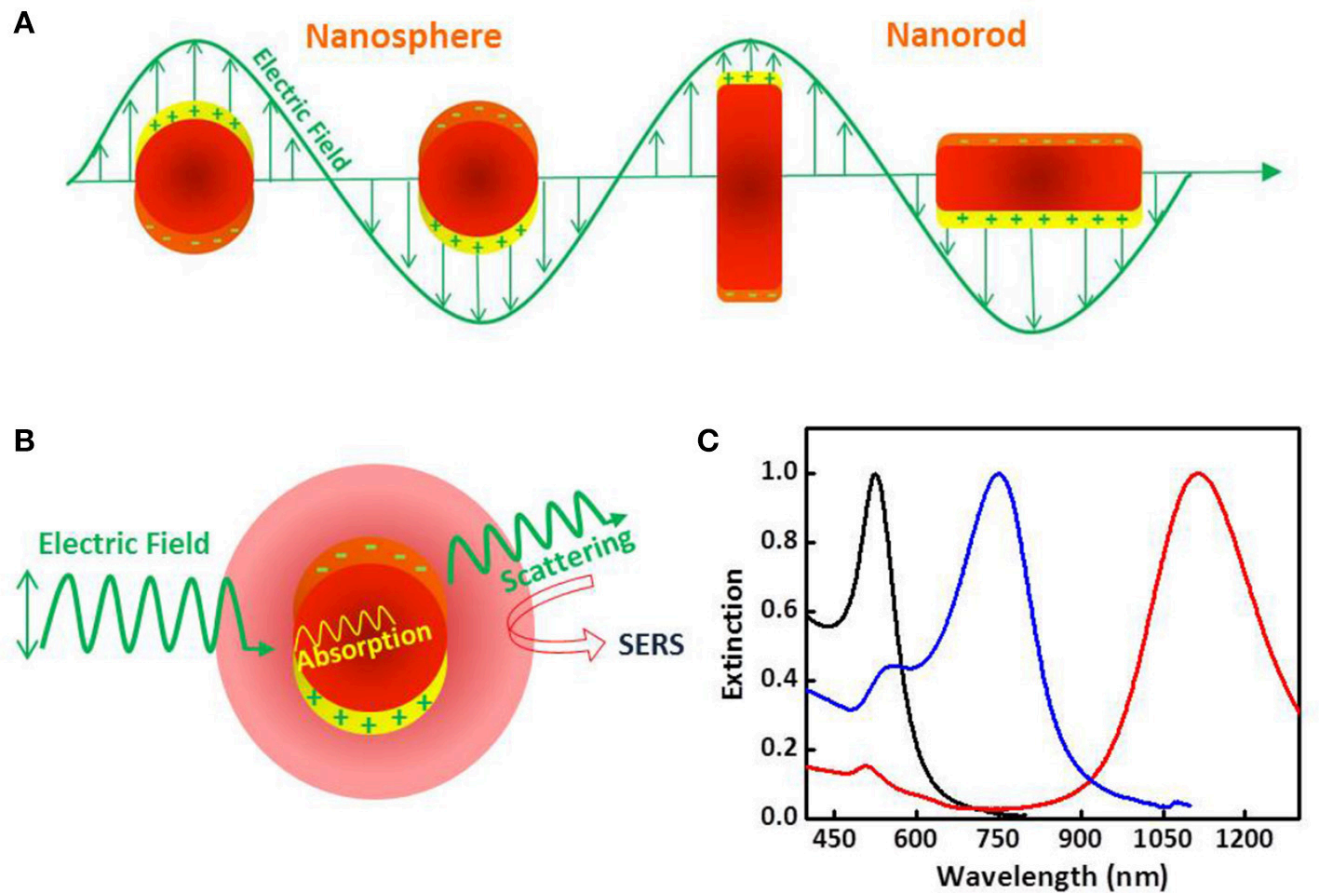
Plasmonic and optical properties of metallic nanoparticles. **(A)** Schematic drawing of the interaction of an electromagnetic radiation with a nanosphere (left) and a nanorod (right). It generates a dipole that oscillates in resonance with the electric field of the incident light as well as a strong confined electric field at the nanoparticle surface. In the case of the metal nanorod two different oscillation modes are observed due to the electron oscillation along the long and short axes. **(B)** Schematic drawing of the two processes, light scattering and absorption, occurring when a metal nanoparticle interacts with light. **(C)** Extinction (scattering + absorption) spectra of gold nanospheres (blak), nanostars (blue) and nanorods (red) in water.

In general terms, the SERS effect can be explained in terms of two enhancement mechanisms; electromagnetic and chemical. The former relies on the generation of high local electromagnetic fields at the surface of metal nanoparticles due to localized surface plasmon resonance (LSPR) excitation, which occurs when conduction electrons collectively oscillate in resonance with the frequency of incident light (Figure 2A). This in turn promotes large enhancements (by many orders of magnitude) of the Raman scattering by adsorbed molecules. Nanoparticle aggregates can provide a significantly larger enhancement due to coupling between LSPRs of the different particles within the aggregate, resulting in higher electromagnetic fields at interparticle gaps within the interacting nanostructures, which are called “hot spots” (Halas et al., 2011). The intense localized fields can interact with molecules in contact with or near the metal surface, typically at distances below 10 nm, so that SERS can be measured (Schlücker, 2014). The chemical mechanism is based on charge transfer processes occurring between the metal nanoparticle and the molecule, but this mechanism has proved to have much lower contribution than the electromagnetic enhancement (Schlücker, 2014; Prochazka, 2016). In addition, the intensity of the Raman scattering signal can be further increased by several orders of magnitude when the frequency of the excitation laser is in resonance with an electronic transition of the molecule, which is known as surface-enhanced resonance Raman scattering (SERRS) (McNay et al., 2011).

Nanoparticles of noble metals, su ch as gold and silver, are optical enhancers of choice in SERS because they resonantly scatter and absorb light in the visible and near-infrared spectral region upon excitation (Figure 2B). The plasmonic properties of these noble metal nanoparticles, namely LSPR and the magnitude of the electromagnetic field generated at the surface, are mainly determined by the nanoparticle size, shape and composition (Figure 2C), and dielectric properties of surrounding medium (Kelly et al., 2003; Yu et al., 2017).

In general, silver is a much more efficient optical transducer than gold, and therefore higher SERS enhancement is to be expected. However, silver displays toxic effects to living organisms, which limits its use for *in vivo* applications. Gold is more chemically inert and robust, and offers better control of its particle size and shape, thereby enabling a wider range of synthetic possibilities, as well as its significantly higher biocompatibility. This is fundamental, since size, shape, composition and stability should be carefully controlled in order to achieve sensitive and reproducible SERS detection. It has been known for a long time that nanoparticle aggregates exhibit larger Raman signal enhancements than individual nanoparticles. This is due to the generation of hot spots within the interparticle gaps. Remarkably, the electromagnetic field enhancement in hot spots is highly sensitive to the detailed local structure and nature of nanoparticle assemblies (Halas et al., 2011), thus top-down lithographic approaches and bottom-up self-assembly methods have been developed to assemble plasmonic nanostructures with precisely controlled geometry and hot spots (Gwo et al., 2016; Mosier-Boss, 2017; Hamon and Liz-Marzán, 2018).

**Figure 3:**
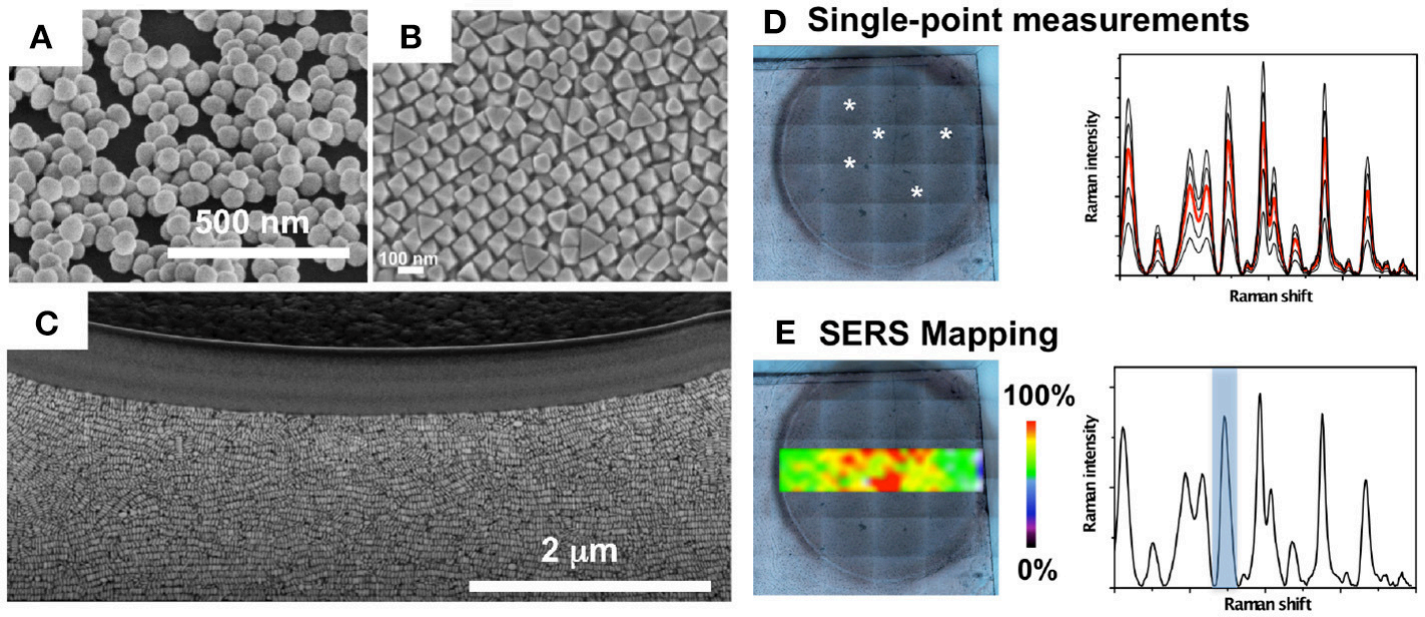
Nanostructured plasmonic substrates for SERS spectroscopy. **(A–C)** Nanostructured plasmonic platforms: Clusters of gold nanoparticles **(A)**, self-assembled gold octahedral nanoparticles **(B)**, and gold nanorods supercrystals embedded in a silica layer **(C)**. **(D)** Single-point SERS measurements on a bacterial colony (white asterisks) grown on a plasmonic nanostructured substrate, and recorded SERS spectra highlighting in red the mean spectrum. **(E)** SERS intensity mapping performed with the spectral peak indicated in the SERS spectrum with a blue bar. **(A,D,E)** Images reproduced with permission from Bodelón et al. (2017). **(B)** Image reproduced with permission from Gómez-Graña et al. (2015). **(C)** Image reproduced with permission from Hamon et al. (2016).

Broadly, two strategies may be followed for direct, label-free, SERS measurements of a biological system (i.e., biomolecule, protein, bacterial cell, biofilm, etc.). The first one involves plasmonic colloids, which are mixed with the sample and the SERS spectra are recorded upon aggregation of nanoparticles. The second approach entails the use of plasmonic platforms based on assemblies of plasmonic nanoparticles and plasmonic patterns over a surface (i.e., nanostructured plasmonic substrates), which offers the possibility to control nanoparticle clustering and the topological parameters of hot spots, leading to improved sensitivity and reproducibility of the SERS measurements. In Figure 3 it is shown different nanostructured platforms bearing increased complexity, from clusters of gold nanoparticles randomly formed on a glass surface, to self-assembled gold octahedral nanoparticles, and gold nanorods supercrystals embedded in a silica layer (Figures 3A–C). This figure also illustrates two measuring modalities for direct SERS detection of metabolites excreted by a bacterial colony grown on the nanostructured plasmonic substrate. In one of them, SERS measurements can be recorded on the plasmonic platform at different points and an average spectrum may be generated (Figure 3D). In the SERS mapping modality (i.e., SERS imaging), a two-dimensional SERS intensity map can be generated in order to visualize the spatial distribution of the detected metabolite on the plasmonic sensor (Figure 3E). For SERS mapping, an area over the substrate is divided into a grid where each square represents a pixel. A series of SERS spectra are acquired at each pixel, and the SERS intensity image (false color) is generated by representing a specific spectral peak of the molecule of interest measured at a fixed wavenumber.

Different parameters such as the excitation laser wavelength and the microscope objective are important aspects that may be considered during SERS. The choice of the excitation laser line depends on the analyte and the optical properties of the plasmonic material. Regarding the analyte, an excitation laser wavelength overlapping or being very close to an electronic transition of the molecule is preferred so as to measure under SERRS conditions. Regarding the plasmonic material, it is important to consider an appropriate wavelength source to enable efficient excitation of the surface plasmons. For nanoparticle suspensions it is predicted that maximum SERS signal can be obtained when the plasmon frequency is tuned to be slightly red-shifted from the laser wavelength. For hot-spot containing plasmonic materials it has been demonstrated that depending on symmetry effects and differences in plasmonic coupling strength the highest SERS intensity could be independent of the excitation source (Sharma et al., 2012). The choice of the microscope objective will determine the spatial resolution of the measurements. The spatial resolution is dependent on the spot size of the illuminating beam, which is dependent on the optics and the wavelength of the laser, leading the higher magnification objectives to the highest spatial resolution. Detailed information regarding the experimental setup of SERS can be found elsewhere (Palonpon et al., 2013; Butler et al., 2016). A typical SERS analysis can accumulate highly complex spectral data sets, by which extraction of chemical and structural information underpinning the biological system is often challenging. For this reason, chemometric analysis such as principal component analysis (PCA), hierarchical cluster analysis (HCA) or partial least squares discriminant analysis (PLS-DA), among others, have become routine in SERS studies. These statistical methods enable to properly evaluate extensive Raman spectroscopic data, and to facilitate reliable identification and potential quantification of the SERS detected chemical features (Cooper, 1999).

## SERS applications in research on quorum sensing in *P. aeruginosa*

Bacteria possess an extraordinary chemical repertoire for intercellular communication and social behavior. Among them, N-acyl homoserine lactones (AHLs) are employed as signaling molecules for many Gram-negative bacteria and have become a paradigm for bacteria intercellular signaling (Papenfort and Bassler, 2016). Different types of AHLs have been identified and characterized in the last decades. In general, they are composed of a homoserine lactone ring with an acyl chain that varies from C4 to C18, which can be slightly modified in some cases by substitution at the C3 position and unsaturation at the C1 position. Once produced they diffuse in and out of the cell and, at a given threshold cell number, they bind to a cognate DNA-binding transcription factor that regulates the expression of QS regulons. The structure and concentration of these molecules play significant roles in the intercellular signaling process (Papenfort and Bassler, 2016). The detection of AHL signal molecules is important not only for gaining new understanding of cell-to-cell communication in live microbial populations, but also because these signaling molecules are involved in the regulation of virulence phenotypes and they have been identified in patients infected with *P. aeruginosa* (Singh et al., 2000). Thus, numerous analytical procedures have been developed for the detection and structural determination of these chemical compounds (Steindler and Venturi, 2007; Wang et al., 2011).

Several approaches employing colloidal suspensions of silver nanoparticles have been applied to determine the viability of SERS to detect AHL signaling molecules. Aggregation of silver nanoparticles is a very common means of achieving strong SERS signals owing to the hot-spots formation and facile preparation. However, this method has traditionally strived with inconsistent measuring and low reproducibility. Following this strategy, Pearman and collaborators detected seven types of commercial AHLs in water. In this study it was shown that the Raman spectra of the different AHLs were highly similar, which hinders the differentiation of signaling molecules by SERS. Among the different AHLs, only 3-oxo-C6-AHL was detected at the relevant biological concentration of 10^−6^ M (Pearman et al., 2016). Likewise, Claussen and collaborators employed silver nanoparticles to detected N-Dodecanoyl-DL-homoserine lactones (C12-AHLs) in spiked culture medium, achieving a detection limit of 0.2 nM (Claussen et al., 2013). This study demonstrated the possibility of SERS for label-free detection of AHLs in bacterial cultures. However, despite these efforts, the SERS detection of natural AHLs produced by bacterial cultures *in situ* has not been achieved yet, most likely due to their low Raman activity. Interestingly, non-enhanced confocal Raman spectroscopy, combined with secondary ion mass spectrometry (SIMS), has been successfully applied in a multimodal chemical imaging approach to evaluate the spatial distribution of quinolone QS molecules across *P. aeruginosa* biofilms throughout various states of organization (Lanni et al., 2014; Baig et al., 2015). The use of SERS for the detection of these signaling molecules remains to be shown.

**Figure 4:**
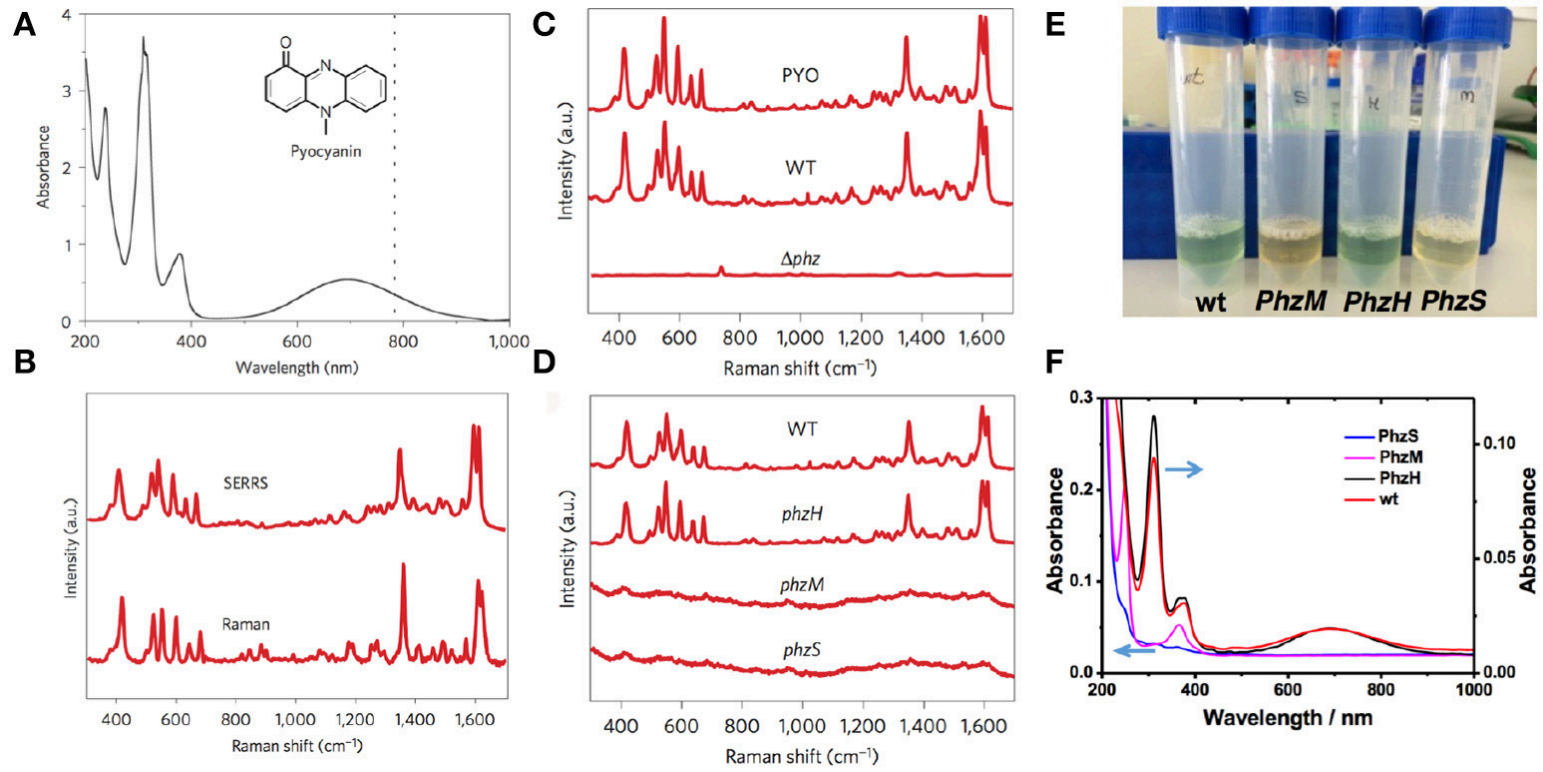
SERRS detection of pyocyanin produced by *P. aeruginosa* PA14 strains grown in planktonic culture. **(A)** UV–visible–near-infrared spectrum of aqueous pyocyanin solution (10^−4^ M) and molecular structure of pyocyanin (inset). The dotted line indicates 785 nm, corresponding to the excitation wavelength used for SERRS. **(B)** Resonance Raman and SERRS spectra of pyocyanin measured in solid state and in aqueous solution (1 μM, Au@pNIPAM hydrogel), respectively. Raman measurement was carried out with a 50× objective, a maximum power of 54.22 kW cm^−2^ and an acquisition time of 10 s. SERRS measurement was carried out with a 20× objective, a maximum power of 4.24 kW cm^−2^ and an acquisition time of 10 s. **(C)** SERRS spectra of commercial pyocyanin (PYO) and of pyocyanin produced by the wild-type (WT) and the phenazine-null *phz1/2* (Δ*phz*) strains. **(D)** SERRS spectra of pyocyanin produced by wild-type and the indicated phenazine mutant strains. **(E)** Photographs of the phenazine-containing samples obtained from the wild type PA14 (wt) and the different mutants (PhzH, PhzS and PhzM), as labeled, under visible light illumination. **(F)** UV-Vis-NIR spectra of the samples containing different phenazines; pyocyanin (wt and PhzH), 1-hydroxyphenazine (1-HO-PHZ, wt, PhzM, and PhzH) and phenazine-1-carboxamide (PCN, wt, PhzS, and PhzM). All SERRS measurements were performed with a 785 nm laser line employing a 20× objective, maximum power between 1.72 kW cm^−2^ and an acquisition time of 10 s (intensity at 418 cm^−1^) employing Au@pNIPAM hydrogels. Images reproduced with permission from Bodelón et al. (2016).

Due to the inherent limitations of SERS, direct detection of target analytes (i.e., microbial metabolites) in complex biological environments still represents a significant challenge. One of these limitations is related to the intrinsic complexity of the biological matrix that may prevent the interaction of the target analyte with the metallic surface. In turn, this would hinder analysis by SERS, as other molecular species interacting with the metal would increase background signal (see limitations and challenges section). In a recent work Bodelón and coworkers developed an approach for label-free SERS detection and imaging of pyocyanin, as a proxy of QS in live biofilm communities of *P. aeruginosa* grown on rationally designed plasmonic substrates (Bodelón et al., 2016). The nanostructured hybrid materials comprised a plasmonic component (i.e., gold nanoparticles) embedded in a porous matrix acting as a molecular sieve for allowing diffusion of small molecules into the underlying optical sensor. The porous nature of the substrates was devised so as to restrict the contact of the plasmonic component with high-molecular weight biomolecules that could otherwise contaminate the SERS spectrum and hinder the sensitivity of the detection. With this in mind and aiming at providing different analytical tools to investigate this form of bacterial communication in live biofilm communities of *P. aeruginosa*, three different cell-compatible plasmonic substrates were fabricated: (1) poly N-isopropylacrylamide (pNIPAM) hydrogel doped with gold nanorods (Au@pNIPAM), (2) mesoestructured Au@TiO_2_ thin film over a layer of gold nanospheres, and (3) micropatterned Au@SiO_2_ supercrystal arrays comprising gold nanorods assembled in micrometer-sized pedestal-like structures coated with a mesoporous silica thin layer. In their study, the authors focused on pyocyanin, a heterocyclic nitrogen containing metabolite that is regulated by QS. Pyocyanin functions as an intercellular signaling molecule in the QS network of *P. aeruginosa* (Dietrich et al., 2006), acts as a virulence factor in infected hosts (Hall et al., 2016), and displays antimicrobial properties against a number of bacterial species (Baron and Rowe, 1981). Taking advantage that pyocyanin exhibits an absorption band in the visible (550–900 nm) the authors employed a 785 nm excitation laser line to increase the Raman scattering signal of the molecule by SERRS (Figures 4A,B). SERRS analysis of cell-free stationary-phase cultures obtained from wild-type PA14 bacteria (WT) grown with constant agitation, showed a SERRS fingerprint almost identical to that of commercial pyocyanin (PYO), whereas no pyocyanin signal was detected in a sample from a phenazine-null mutant strain (Δ*phz*) (Mavrodi et al., 2001; Figure 4C). The SERRS fingerprint is pyocyanin-specific since it is not detected in stationary-phase cultures of PA14 mutant strains Δ*phzM* and Δ*phzS* (Mavrodi et al., 2001), which are deficient in the biosynthesis of this phenazine (Figure 4D). The measurement under resonance Raman conditions facilitates the selective detection of pyocyanin over the rest of the phenazines produced by PA14 bacteria because they lack the 550–900 nm absorption band (Figures 4E,F; Bodelón et al., 2016).

**Figure 5:**
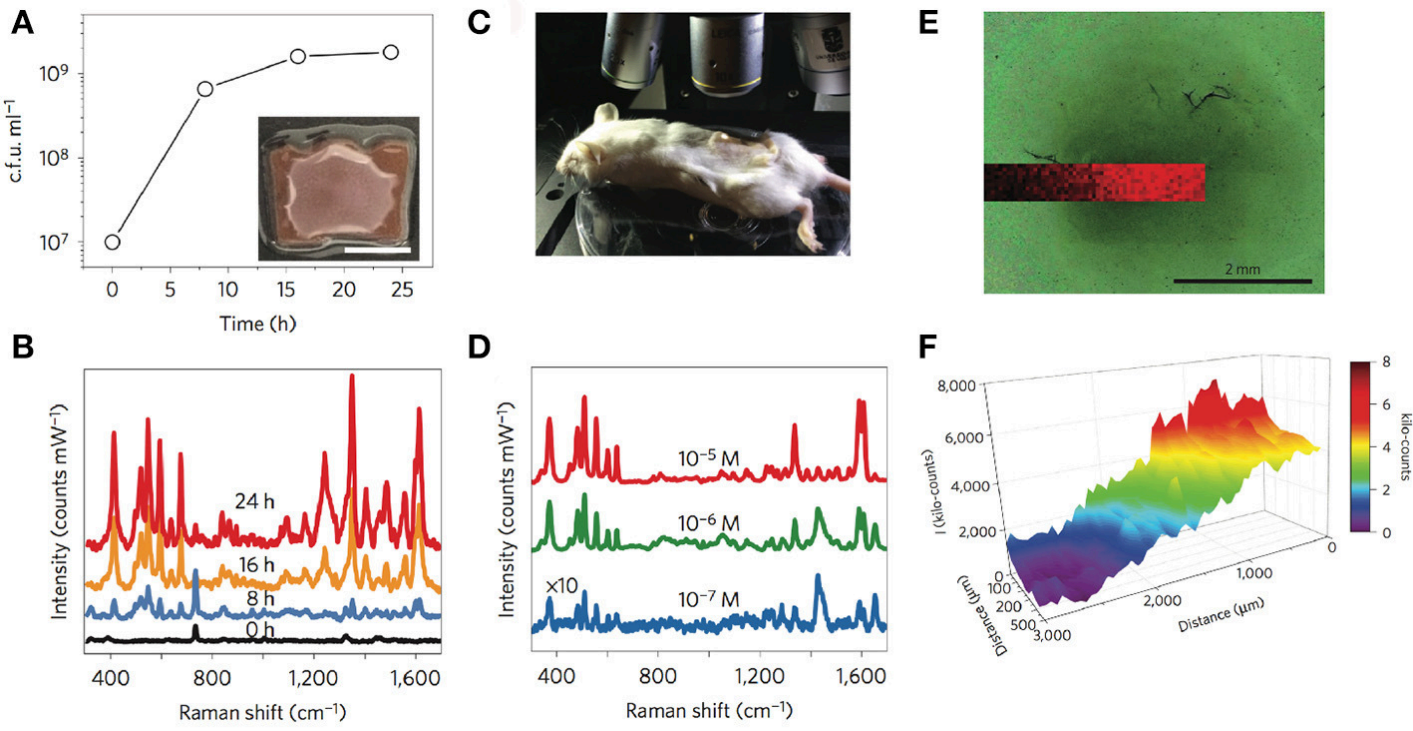
*In situ* detection and imaging of pyocyanin secreted by *P. aeruginosa* PA14 colonies and biofilms grown on Au@pNIPAM hydrogels and mesoestructured Au@TiO_2_ thin films. **(A)** Graphical representation of viable bacteria (c.f.u. ml^−1^) quantified over time. The inset shows an image of the colony-biofilm grown on Au@pNIPAM (scale bar, 0.5 cm). **(B)** SERRS spectra recorded at the indicated times. Measurements of colony-biofilms were done using a 785 nm laser line for 10 s and using a maximum power of 0.91 kW cm^−2^ employing a 20× objective. **(C)** Photograph showing the Raman experimental set-up for detection of pyocyanin in subcutaneous implants in mice. **(D)** Under-skin SERRS spectra of pyocyanin spiked at the indicated concentrations on Au@pNIPAM hydrogel. SERRS measurements of pyocyanin-spiked hydrogels were performed using a 785 nm laser line for 10 s using a maximum power of 24.45 kW cm^−2^ employing a 10× objective. For clarity, the spectra noted with × 10 have been multiplied by a factor of 10. **(E)** Optical image of bacterial biofilm (dark central region) grown on Au@TiO_2_ substrate captured with the Raman microscope and superimposed pyocyanin SERRS mapping (418 cm^−1^) acquired with excitation laser wavelength of 785 nm, 5× objective and a laser power of 0.94 mW for 10 s. **(F)** Graphical representation of the SERRS intensity mapping shown in **(E)**. Images reproduced with permission from Bodelón et al. (2016). Copyright © 2016, Springer Nature.

The authors demonstrated quantitative SERRS detection of pyocyanin in a concentration range between 0.1 μM down to 1 nM in aqueous samples obtained from chloroform extracted *P. aeruginosa* culture supernatants, achieving limits of detection (LOD) ranging from 10^−10^ M for Au@pNIPAM hydrogel, 10^−9^ M for mesoporous Au@TiO_2_ thin film and 10^−14^ M for the micropatterned mesoporous Au@SiO_2_ substrate. Interestingly, the hybrid plasmonic substrates were shown to facilitate *in situ* SERRS detection of pyocyanin produced by biofilms and small cellular aggregates of *P. aeruginosa* grown in droplets, and yielded spatially resolved 2D maps of the QS molecule with high spatial resolution. The Au@pNIPAM hydrogel, devised as a highly porous platform with enhanced diffusivity, led to plasmonic detection of pyocyanin throughout the growth of the colony-biofilm (Figures 5A,B) with a homogeneous distribution in both colonized and non-colonized regions of the substrate. Interestingly, the 785 nm near-infrared laser enabled to detect this metabolite at biologically relevant concentrations (i.e., as low as 0.1 μM) in spiked Au@pNIPAM hydrogels implanted subcutaneously in mice (Figures 5C,D), indicating that pyocyanin could be used as a reporter for non-invasive monitoring of QS and screening potential antimicrobial drugs in animal models of infections using SERRS (Bodelón et al., 2016). Indeed, the expression of pyocyanin is a common phenotypic assay widely used in quorum quenching studies as an indicator of the efficacy of the treatment. In view of these results, the authors suggested that plasmonic hydrogels could be used as implantable materials in experimental animal models, to investigate QS triggered by natural populations of *P. aeruginosa* and to assess anti-virulence therapies by SERS (Bodelón et al., 2016). Imaging QS in live biofilms with spatiotemporal resolution is important toward gaining new understanding of this form of bacterial communication. In this work, it was demonstrated spatial imaging of pyocyanin produced by biofilms of *P. aeruginosa* PA14 grown on mesostructured Au@TiO_2_ thin films with a resolution of about 20 μm, as well as variation of the QS signal up to millimeter-scale areas (Figures 5E,F).

**Figure 6:**
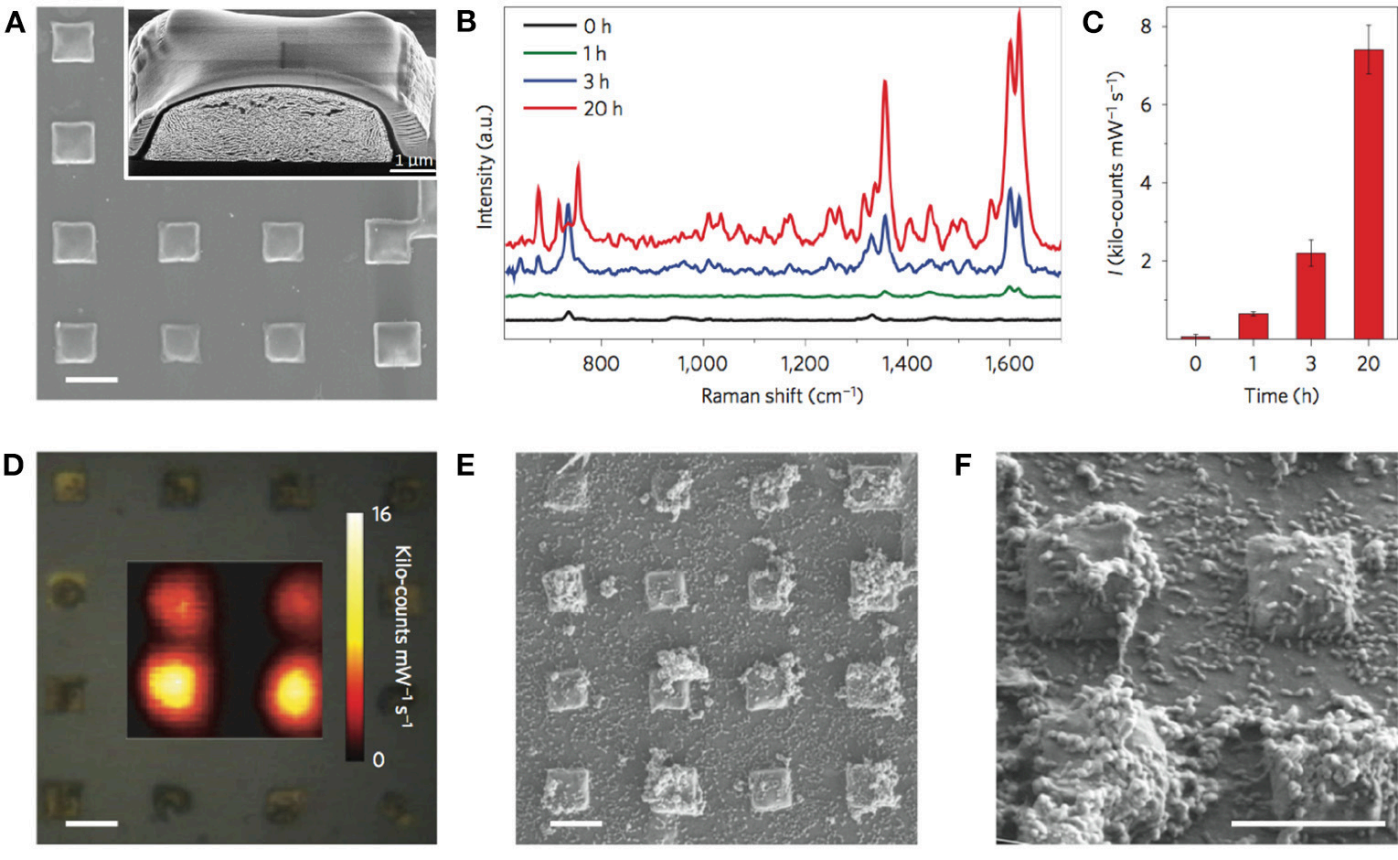
*In situ* detection and imaging of pyocyanin secreted by *P. aeruginosa* PA14 biofilms grown on micropatterned Au@SiO_2_ supercrystals. **(A)** SEM images of micropatterned Au@SiO_2_ supercrystals. Scale bar, 5 μm. The inset shows a cross-section SEM image of a Au@SiO_2_ supercrystal. **(B)** Representative SERRS spectra measured at 0, 1, 3, and 20 h. **(C)** Relative SERRS intensities (1,600 cm^−1^) recorded at 0, 1, 3, and 20 h. Error bars indicate standard deviation. **(D)** Optical image of the substrate and SERRS mapping of pyocyanin (1,600 cm^−1^) recorded at 20 h of growth. Scale bar, 5 μm. **(E,F)** SEM images of Au@SiO_2_ supercrystals colonized by *P. aeruginosa* (20 h) at different magnifications. Scale bars, 5 μm. All SERS measurements were carried out with a 785 nm laser line, 50× objective and a maximum power of 0.98 kW cm^−2^. The acquisition time was 0.1 s. Images reproduced with permission from Bodelón et al. (2016).

Owing to its extremely high enhancement factor toward pyocyanin detection (LOD 10^−14^ M), the Au@SiO_2_ supercrystal platform enabled ultrasensitive SERRS detection of pyocyanin in low-density bacterial cultures at early stages of biofilm development, and imaging of bacterial communication triggered by small clusters of cells colonizing the micrometer-sized plasmonic features (Figure 6). The high performance of this substrate was most likely due to a high density of efficient hot-spots and collective plasmon modes in the supercrystal, as well as a contribution of the mesoporous silica coating, which infiltrates within the highly ordered structure of nanorods, thereby increasing the “plasmonically active space” and leading to an extremely high electromagnetic enhancement factor (Hamon et al., 2014, 2016). The SERS-based approach employed by Bodelón and collaborators, focusing on the detection of pyocyanin released from bacterial biofilms and small clusters of cells, demonstrated the potential of plasmonics as an alternative method for non-invasive detection and imaging SERS-active metabolites released from undisturbed microbial populations. For diagnostic purposes, ultrasensitive SERRS detection of pyocyanin at trace levels could aid in early detection and effective treatment of *P. aeruginosa* infectious disease.

Multidrug resistance is an increasing threat to the successful treatment of bacterial infections. In particular, *P. aeruginosa* has the ability to rapidly develop resistance to multiple classes of antibiotics leading to high morbidity and mortality rates (Rossolini and Mantengoli, 2005). Early detection and timely administration of antimicrobial therapy is critical in optimizing patient outcomes, including hospital length of stay, mortality, and healthcare costs. Therefore, sensitive and reliable methods for rapid microbial identification are essential in modern healthcare (Bauer et al., 2014; Cookson et al., 2017). An alternative approach relies in the identification of the infectious agent based on the detection of pathogen-specific biomarkers. In this context, Hunter and collaborators demonstrated a correlation between pyocyanin concentration in sputum and rates of pulmonary decline in adult patients with CF chronically infected with *P. aeruginosa*, indicating that this metabolite can serve as an important diagnostic indicator (Hunter et al., 2012). The detection and quantification of pyocyanin in sputum was determined by high performance liquid chromatography, an analytical technique with limited throughput that requires substantial expertise and know-how. As an alternative diagnostic analytical method, Wu and collaborators implemented a SERS-based approach employing silver nanorod arrays for detecting pyocyanin in processed (i.e., chloroform-extracted) clinical sputum samples. The system allowed the detection of the metabolite at clinically relevant concentrations with the advantage to process multiple samples rapidly (Wu et al., 2014).

**Figure 7:**
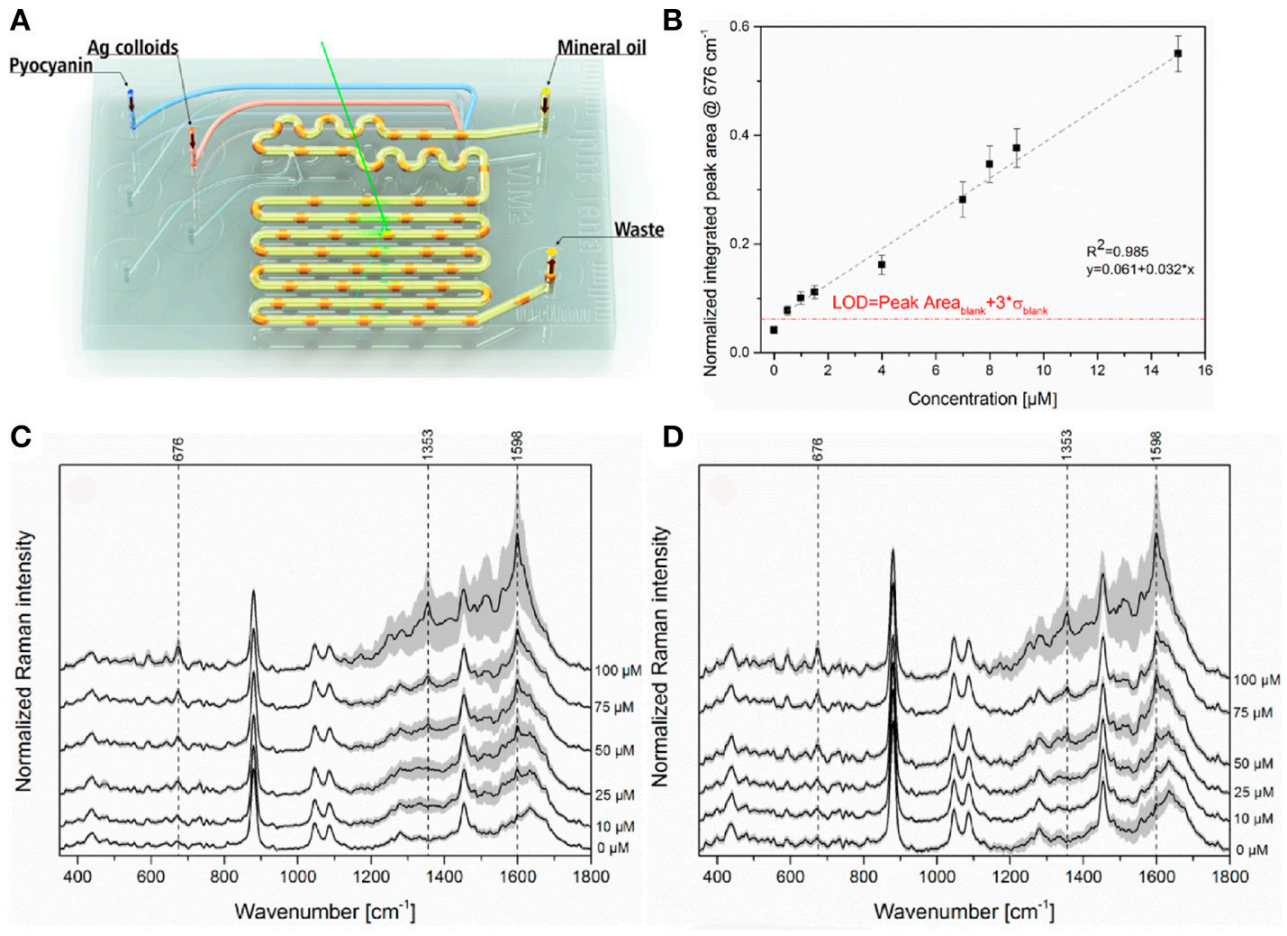
LoC-SERS approach for detection of pyocyanin in saliva. **(A)** Schematic representation of the droplet based microfluidic chip used for LoC-SERS measurements. **(B)** Calibration curve for the determination of pyocyanin relative concentration in aqueous solution. The peak area ratio of the 676 cm^−1^ and 240 cm^−1^ Raman modes as a function of pyocyanin concentration in aqueous solution in the range between 0.5 and 15 μM with linear fitting. The red line indicates the calculated LOD. **(C,D)** Average SERS spectra and their double standard deviation of the different concentrations of pyocyanin in the saliva sample from volunteer number one **(C)**, two **(D)**. Images reproduced with permission from Žukovskaja et al. (2017).

Recent advances in microfabrication technologies have made it possible to obtain microscale devices for culturing microbial cells (Weibel et al., 2007), which have the capability not only to transform the study of microbial physiology and cellular communication, including QS, but also hold great potential for many practical applications including drug discovery and diagnosis (Srinivasan et al., 2015; Nai and Meyer, 2017). The success of this emerging field requires the adaptation of sensitive analytical tools able to detect trace amounts of target biomolecules, an application for which SERS has great potential. In this respect, Žukovskaja and collaborators developed a lab-on-a-chip SERS (LoC-SERS)-based microfluidic system (Figure 7), which was applied to detect pyocyanin spiked in saliva at the clinical micromolar range employing silver colloids without the need of sample processing (Žukovskaja et al., 2017).

**Figure 8:**
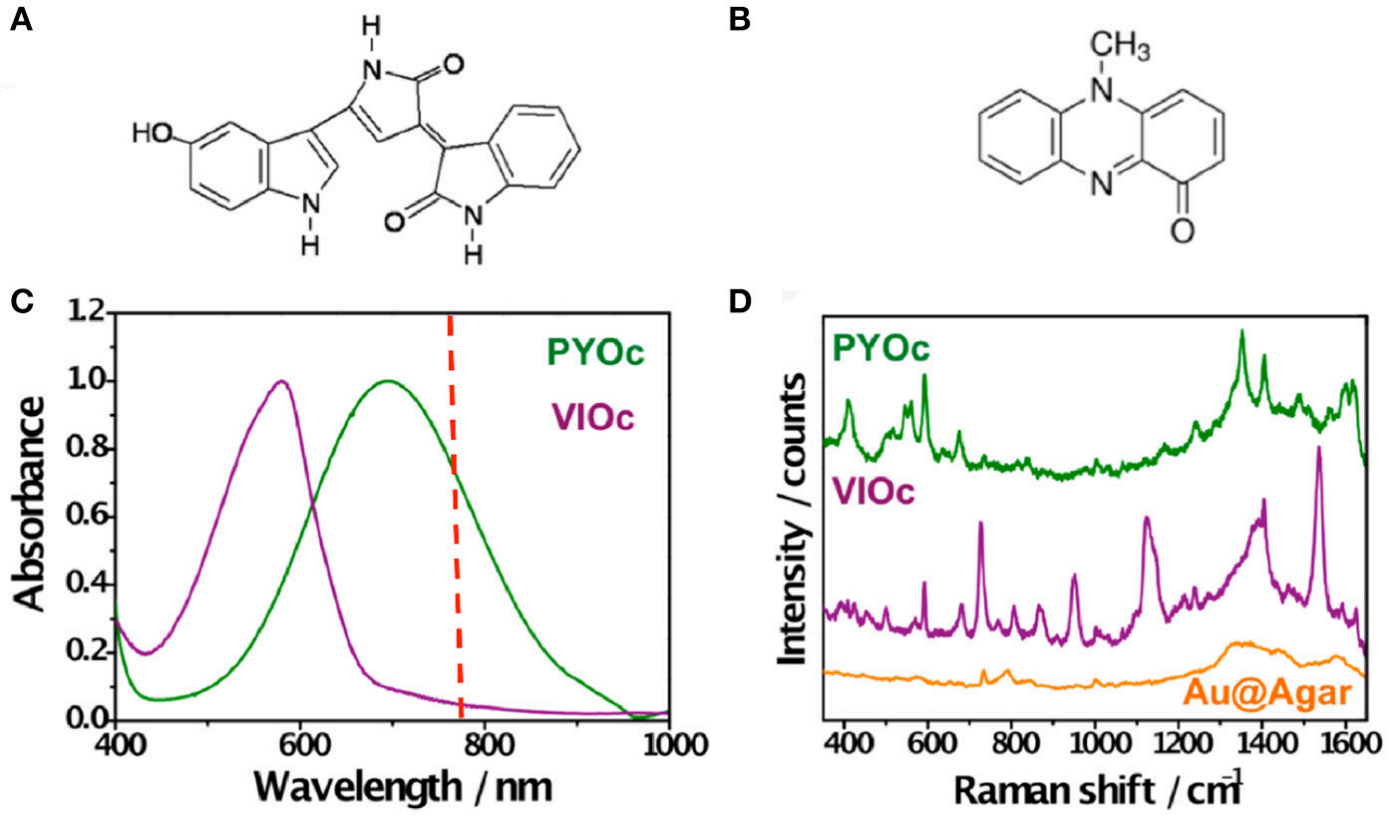
Detection of violacein and pyocyanin on Au@agar by SERS/SERRS. **(A,B)** Chemical structures of violacein and pyocyanin, respectively. **(C)** Normalized visible–NIR spectra of commercial pyocyanin (PYOc) and violacein (VIOc). The dashed red line indicates the laser wavelength used (785 nm). **(D)** SERRS spectrum of PYOc, SERS spectrum of VIOc, and SERS spectrum of Au@agar. All spectra were measured with a 50× objective, a maximum power of 0.64 kWcm^−2^, and an acquisition time of 10 s. Excitation laser line was 785 nm. Images reproduced with permission from Bodelón et al. (2017).

In an effort to extend the use of SERS as a imaging tool to study interspecies QS communication, Bodelón and collaborators demonstrated the simultaneous detection of pyocyanin and violacein produced by interacting colonies of *P. aeruginosa* PA14 and *Chromobacterium violaceum* CV026, respectively, grown as a co-culture on agar-based hybrid nanostructured plasmonic (Au@agar) substrates (Bodelón et al., 2017). This platform comprises a multilayer thin film of gold nanospheres on glass covered by a thin layer of nutrient LB agar. The motivation behind the use of a solid culture medium (e.g., agar-based) is that it enables co-culturing of microbial colonies at predefined locations with controlled separation. By confronting microbial populations on agar, the microorganisms can be readily identified as discrete colonies, as well as the region of chemical interaction between them. In the study, *P. aeruginosa* PA14 and *C. violaceum* CV026 were selected as a dual species co-culture model because the QS systems of these soil saprophytic bacteria are known to regulate the biosynthesis of pyocyanin and violacein, respectively, molecules amenable to Raman spectroscopy detection most likely due to their high Raman cross-section. *P. aeruginosa* produces two types of AHL QS signaling molecules: C12-AHL and N-butyryl-L-homoserine lactones (C4-AHLs) that are involved in the production of pyocyanin (Lee and Zhang, 2014). CV026 is a mutant strain of *C. violaceum* that cannot generate its own AHL signals, but can respond to compatible AHLs bearing short C4 to C8 acyl chains, such as *P. aeruginosa* C4-AHLs, thereby resulting in the expression of QS-regulated phenotypes, including the synthesis of violacein (McClean et al., 1997). The precise biological function of this pigmented metabolite still remains to be elucidated, but it has been shown to display toxic activities against certain bacterial species and predator organisms (Durán et al., 2016). As violacein and pyocyanin possess absorption bands centered at 580 and 695 nm, respectively, the use of a 785 nm excitation laser line enabled SERS detection of violacein and SERRS detection of pyocyanin (Figure 8).

**Figure 9:**
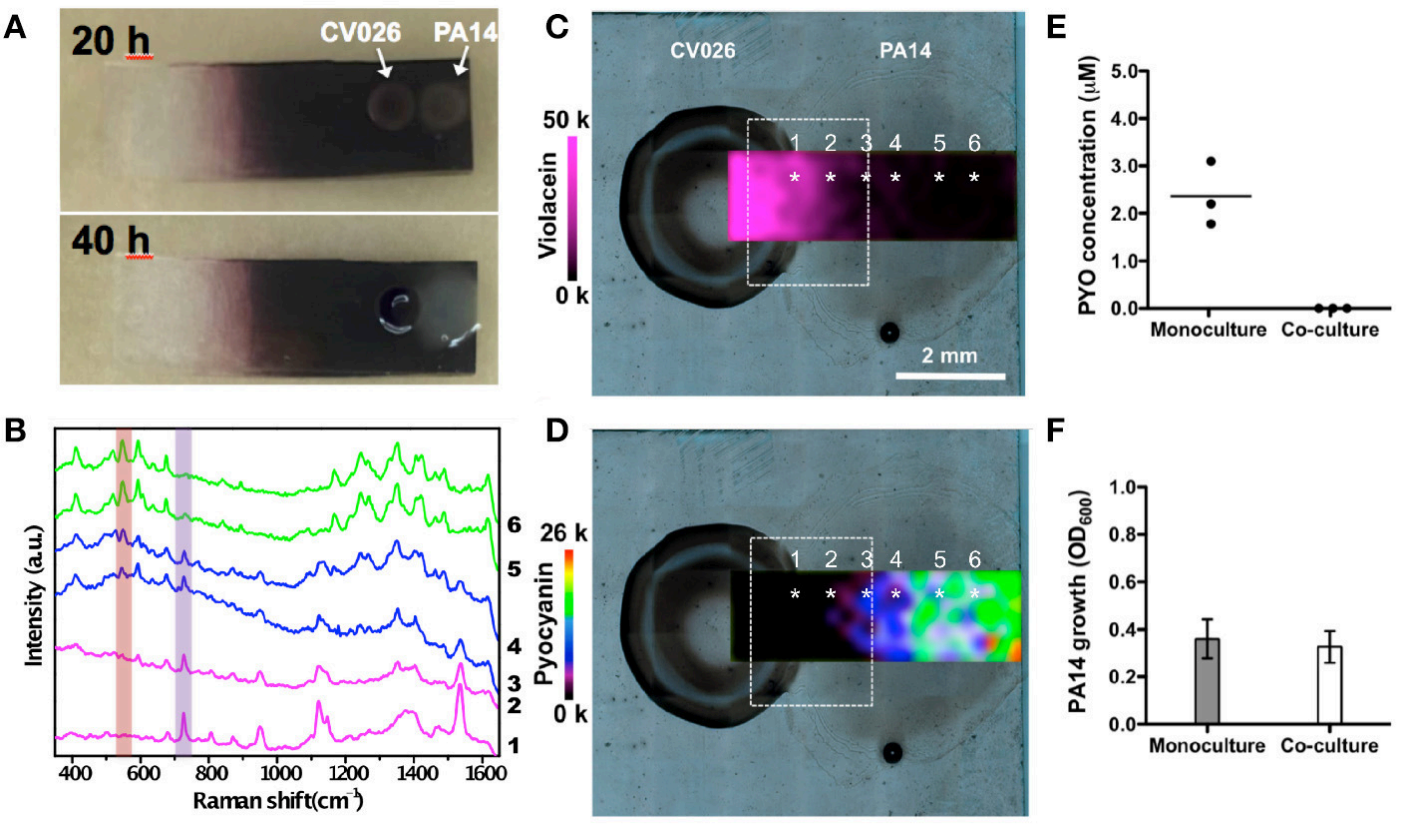
Detection and imaging of interspecies QS on Au@agar by SERS/SERRS. **(A)** Photographs of *C. violaceum* CV026 and *P. aeruginosa* PA14 colonies co-cultured on Au@agar taken at 20 and 40 h. **(B)** SERS/SERRS spectra recorded at the points indicated with asterisks in **(C,D)**. The purple and orange bars indicate violacein (727 cm^−1^) and pyocyanin (544 cm^−1^) specific bands. **(C,D)** SERS mapping of violacein (727 cm^−1^) **(C)** and SERRS mapping of pyocyanin (544 cm^−1^) **(D)** in co-culture at 20 h. The dashed squares indicate the confrontation zone. **(E)** Quantification of pyocyanin (PYO) produced by PA14 colonies grown in monoculture or in co-culture by UV-Vis spectroscopy. Dark circles indicate the value obtained from biological triplicates (*n* = 6, for each group). Straight line within the data points indicates average. **(F)** Growth of PA14 bacterial cells in monoculture and in co-culture. Error bars indicate the standard deviation of biological triplicates. Images reproduced with permission from Bodelón et al. (2017).

Initially, the detection of violacein expression was demonstrated by SERS in CV026 bacterial cells grown as a colony on Au@agar upon treatment with commercial C4-AHL. The high sensitivity of the plasmonic approach was demonstrated by the detection of violacein spectral features in non-pigmented CV026 colonies stimulated with a low concentration of commercial C4-AHL. Interestingly, the levels of pyocyanin expression observed by SERRS in co-culture were significantly lower than those in monoculture. Moreover, in co-culture (Figures 9A,B), the amount of pyocyanin and violacein detected were inversely proportional in the confrontation zone (Figures 9C,D), suggesting a possible role of violacein in the down-regulation of the phenazine. To confirm the above data, the phenazine concentration was measured by UV–vis spectroscopy at 691 nm (λmax of pyocyanin) following chloroform extraction from the agar on which the PA14 colonies were grown. Significantly, whereas the amount of pyocyanin released by PA14 cells in monoculture averaged 2.3 μM, its concentration could not be determined in co-culture, as it was below the detection limits of this method (Figure 9E). Since the growth of PA14 bacteria in monoculture and co-culture was very similar (Figure 9F), the differential expression of pyocyanin was not attributed to growth defects.

Notably, quantitative PCR analysis of gene expression indicated that the decreased levels of pyocyanin were, at least in part, due to the repression of the *P. aeruginosa phzS* gene responsible for the last step of pyocyanin biosynthesis. Treatment of PA14 bacteria with commercial violacein reduced pyocyanin expression, as well as and transcription of the *phzS* gene, which indicated a potential role of violacein in the down-regulation of the phenazine. Interestingly, violacein is a bis-indole compound, and it has been reported that indole and its derivatives have been shown to repress QS-regulated phenotypes in *P. aeruginosa* (Lee et al., 2015), including the production of pyocyanin (Chu et al., 2012). Remarkably, PA14 strains expressing pyocyanin (i.e., wild type and Δ*phzH*), as opposed to strains deficient in the biosynthesis of this phenazine (i.e., Δ*phz1/2*, Δ*phzM*, and Δ*phzS*), compromised the growth of CV026. Therefore, this experimental evidence indicated that pyocyanin exerted a toxic effect to *C. violaceum*. As stated by the authors, although PA14 and CV026 bacteria can initially coexist, *P. aeruginosa* eventually overgrows *C. violaceum*, reducing the viability of its partner in extended co-cultures. In view of these results, it was suggested that the promiscuous CviR transcriptional receptor of CV026 can sense C4-AHLs produced by PA14 bacteria producing violacein, which in turn may contribute to pyocyanin down-regulation. This hypothesis points toward a potential defensive mechanism of *C. violaceum* CV026 in the chemical interplay between the bacterial species. This study illustrates the potential of SERS for non-invasive chemical analysis of microbial interactions on agar, which is the standard support matrix for culturing microbial cells, enabling to visualize the expression of two microbial metabolites in the co-culture taking place as a result of QS interspecies communication. In the context of polymicrobial diseases, similar SERS-based approaches could be applied for studying clinically relevant interactions between *P. aeruginosa* and other microbial species such as *S. aureus, Burkholderia* spp, *C. albicans*, etc.

*P. aeruginosa* is a versatile bacterium that has evolved a set of regulatory mechanisms to adapt to nutritional changes and thrive in hostile environments. Recent studies have shown that carbon source has a high impact on bacterial QS signaling, virulence, biofilm formation and pyocyanin production (Shrout et al., 2006; Huang et al., 2012). In this context, Polisetti and collaborators used SERS to image the production of pyoacyanin in pellicle biofilms of a CF clinical strain (FRD1) or a laboratory strain (PAO1C) of *P. aeruginosa* grown in the presence of glutamate or glucose as carbon sources (Polisetti et al., 2016). In this study, silver nanoparticles (12–14 nm) were incubated with biofilms and used as SERS optical enhancers to spatially map pyocyanin by confocal Raman microespectroscopy. For conducting SERS mapping, biofilm samples were deposited onto silicon wafers and dried. A PCA multivariate statistical approach was implemented in the analysis so as to accurately integrate the SERS spectral data acquired from the highly heterogeneous biological matrix. The analysis showed a relatively homogeneous distribution of pyocyanin in biofilms of the CF clinical strain when grown with glucose and glutamate, while the laboratory strain only produced detectable levels of pyocyanin when glutamate was used as the carbon source, thereby demonstrating strain-level differences in carbon metabolism. In addition to pyocyanin, SERS analysis of biofilms from CF clinical strain showed a spectral feature that may correspond to vibrational bands of alginate carbohydrates, associated by the authors to the mucoid phenotype specific of this strain. Mucoid isolates of *P. aeruginosa* are highly prevalent in the CF lung, and their emergence during the course of infection is associated with increased inflammation, respiratory decline, and poor prognosis for CF patients (Koch, 1993).

It has long been known that QS-regulated factors influence different stages of biofilm formation, including cell attachment, growth and dispersal (Passos da Silva et al., 2017). Moreover, the densely populated environment within the biofilm facilitates intercellular chemical interactions and QS communication (Parsek and Greenberg, 2005; Flemming et al., 2016). SERS has been applied for *in-situ* chemical analysis of biofilms, as well as to evaluate the spatial biodistribution of biofilm matrix components and their relative abundance, which has been recently reviewed. (Ivleva et al., 2017) Ivleva and collaborators employed silver nanoparticles as SERS optical enhancers to investigate the matrix components in multispecies biofilms (Ivleva et al., 2008, 2010). Their plasmonic approach led to a significant enhancement of the Raman signals that enabled them to chemically image biofilm matrix constituents. SERS, in contrast to non-enhanced (i.e., conventional) Raman spectroscopy, can help to harness chemical information of the biofilm matrix in more detail, especially at low cell densities (Ivleva et al., 2010). It should be noted that the biotoxicity associated to silver nanoparticles and silver ions may give rise to potential artifacts that could hamper the analysis of the biofilm under *in vivo* conditions (Ivleva et al., 2017). Chao and Zhang also employed silver nanoparticles to investigate chemical variations in the matrix of biofilms of various Gram-negative and Gram-positive bacteria including *Escherichia coli, Pseudomonas putida*, and *Bacillus subtilis*. In this study, biofilms cultivated for 4, 8, 24, and 72 h were incubated with the silver nanoparticles and dried before SERS analysis. By assigning peaks of averaged SERS spectra into the different components of the biofilm matrix, the authors showed that the lipid, nucleic acid, and protein content increased significantly in growing biofilms (Chao and Zhang, 2012). Interestingly, the authors hypothesized that the significant increase during biofilm growth of a predominant Raman band at 730 cm^−1^ assigned to nucleic acids, could be attributed to the accumulation of extracellular DNA. In certain bacterial species such as *P. aeruginosa*, the release of this major structural component of the biofilm matrix is induced by lysis of a bacterial subpopulation in response to QS (Ibáñez de Aldecoa et al., 2017). These studies illustrate the potential of SERS to chemically monitor biofilm microbial communities and provide new insights regarding their structural and spatial organization.

**Figure 10:**
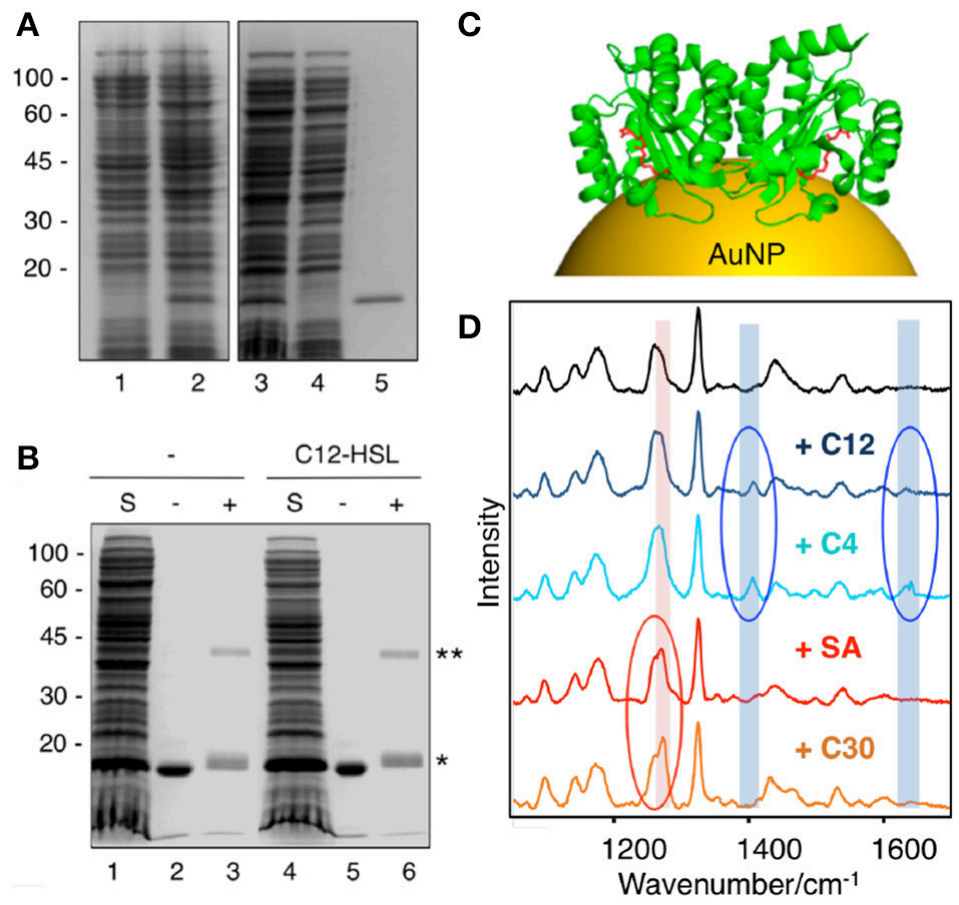
Application of SERS for detection of QS protein-ligand interactions. **(A)** SDS-PAGE and Coomassie blue staining of LasR_LBD_ expression and purification. Lane 1, uninduced total fraction; lane 2, IPTG-induced total fraction; lane 3, IPTG-induced soluble fraction; lane 4, affinity chromatography flow-through; lane 5, eluted protein. **(B)** Cross-linking assay of LasR_LBD_. The polypeptide expressed in bacteria in the absence (lanes 1-3) or in the presence (lanes 4–6) of C12-HSL was solubilized (S), affinity purified and subjected to crosslinking (+) with 0.5 mM DSS (lanes 3,6) or not (lanes 2,5). Bands corresponding to dimeric (^**^) and monomeric (^*^) forms are indicated on the right. **(C)** Schematic illustration of dimeric LasR_LBD_ bound to C12-AHL (in red) (PDB:2UV0) attached to a gold nanoparticle (AuNP). **(D)** SERS spectra (after baseline correction) of LasR_LBD_ polypeptide (black spectrum), incubated with C12-AHL (+C12), C4-AHL, salicylic acid (+SA) or furanone C30 (+C30). The red and blue ovals indicate inhibitor-specific and activator-specific SERS fingerprints, respectively. Illumination with a 785 nm laser line was used to avoid protein damage. Images reproduced with permission from Costas et al. (2015).

Biochemical and functional analysis have shown that most QS LuxR family members require appropriate AHL molecules to properly fold into their active conformations, which is mostly based on the production of soluble and stable protein upon supplementing the bacterial growth medium with cognate signaling molecules. This strategy has been applied toward the structural characterization of several LuxR homologs (Papenfort and Bassler, 2016), including the ligand-binding domain (LBD) of LasR from *P. aeruginosa* (Bottomley et al., 2007). Resolution of their crystal structures have enabled researchers to design and identify chemical compounds capable of binding to the ligand-binding pockets of LuxR-type receptors so as to develop potent QS inhibitors (LaSarre and Federle, 2013). However, the failure to express LuxR homologs in the apoprotein form (i.e., ligand-free) at the high concentrations required for structural characterization has limited the understanding of the mechanisms by which QS receptors are modulated by native and non-native ligands. Taking advantage that certain LuxR homologs, such as LasR from *P. aeruginosa*, can fold into an active conformation in the absence of their cognate AHL ligands (Sappington et al., 2011), Costas and collaborators implemented a SERS-based approach to detect interactions between the LBD of LasR and QS agonists and antagonists (Costas et al., 2015). To this end the LBD of LasR (LasR_LBD_) bearing a hexa-histidine tag and a cysteine in its carboxyl-terminus was expressed and affinity-purified in a soluble, ligand-free active form. By chemical crosslinking of purified LasR_LBD_ with disuccinimidyl suberate (DSS) authors demonstrated the presence of dimeric complexes of LasR_LBD_ at similar levels regardless of the presence or absence of its cognate C12-AHL ligand. This indicates that the polypeptide can exist in the form of homodimers even when it is expressed in the absence of cognate signal molecules (Figures 10A,B). Costas and collaborators showed that apo LasR_LBD_ can bind C12-AHLs, which acted as quorum quencher in a QS reporter system, demonstrating that the apoprotein is competent for ligand-binding. For SERS analysis, LasR_LBD_ was attached to the plasmonic sensor via the thiol group of the carboxy-terminal cysteine and incubated with cognate C12-AHL ligands, C4-AHL agonists and Furanone C30 or acetylsalicylic acid antagonists. Label-free SERS allowed the authors to detect conformational changes of LasR_LBD_ as a result of its interaction with the different QS ligands. The highly sensitive and reproducible SERS spectra allowed the discrimination between activators and inhibitors of QS, through their distinctive vibrational signatures (Figure 10C). This study features SERS as a fast and cost-effective tool to analyze ligand-induced conformational changes in proteins, confirming the applicability of SERS for *in vitro* screening of QS modulators. In this framework, this SERS strategy has great potential to be implemented in structure-activity relationship studies for pharmacophore generation of inhibitors targeting bacterial virulence and antibiotic resistance mechanisms linked with QS.

## Limitations and challenges

Our understanding of SERS mechanisms and the ability to engineer plasmonic nanostructures has increased enormously during the last decade. Researchers have mastered the fabrication of rationally designed plasmonic transducers with tunable optical properties, large SERS enhancement factors, and appropriate surface functionalization (Hamon and Liz-Marzán, 2018), which has allowed to apply this technique with great success in the analytical field to detect a wide range of chemical species at ultralow (i.e., attomolar) concentrations (Wang and Kong, 2015; Mosier-Boss, 2017). However, the implementation of plasmonic transducers for label-free sensing and imaging applications in complex biological environments is still a challenging task.

One of the main hurdles that must be overcome is that the target analyte must be in contact with the plasmonic surface and often has to compete with metal surface ligands and biomolecules, which are usually present in biological media at much higher concentrations. Detecting target molecules with no or low affinity for the metal surface may also represent a significant problem. Different strategies can be applied in order to overcome these potential issues. In general terms, the surface chemistry of nanoparticles may be tailored to improve binding selectivity and facilitate detection. Materials with selective porosity (López-Puente et al., 2013; Bodelón et al., 2016), non-fouling surfaces (Sun et al., 2015), and tunable charge (Jia et al., 2016), offer attractive alternatives by providing chemical or physical filtering of interfering molecules. Due to the fact that SERS is influenced by the nature of the interactions between molecules and nanostructured surfaces, the charge properties and functional groups of molecules and components of the plasmonic substrate play an important role in SERS analysis. SERS performance can be significantly improved upon minimizing electrostatic repulsion forces, as well as by tuning the dielectric (hydrophilic/hydrophobic) properties of the surface, which can be a suitable strategy to trap non-polar molecules (Abalde-Cela et al., 2010). These strategies reduce the need for sample pretreatment, improve selectivity, and can be applied for *in-situ* analysis.

As discussed above, SERS measurements are greatly influenced by the affinity between biomolecules and the metal surface, thereby label-free SERS analysis of target analytes in biological samples (i.e., cells, biofilms) may be dominated by vibrational bands originating from other “contaminating” biomolecular species, which may lead to complex SERS spectra. In this context, the Raman spectrum of the cell and the extracellular medium is characterized by many different vibrational modes of biomolecules, including nucleic acids, proteins, lipids, and carbohydrates, representing a complete biomolecular profile, making the interpretation of the SERS spectrum a challenge for most adsorbates. In order to maximally exploit the capabilities of SERS in microbiology for *in situ* identification, it is essential to understand the molecular and corresponding biochemical origins of SERS vibrational signatures. Researchers have identified the spectral fingerprints of numerous types of biomolecules and molecular constituents, such as lipids, proteins, nucleobases, pigments and certain metabolites. However, despite some efforts (De Gelder et al., 2007), comprehensive databases of SERS and Raman spectra of biomolecules are still needed, and band assignment for the acquired spectra requires the analysis of already published data. Interpretation of the Raman spectra demands data processing as well as statistical treatments such as multivariate data analysis, which is favored by the high resolution of the SERS spectra. In this framework, chemometric pattern recognition algorithms are widely applied in SERS studies so as to improve the accuracy and reproducibility of the technique facilitating, for instance, the monitoring of different extracellular metabolites in the culture medium (Mishra et al., 2017), the discrimination of five types of penicillin G antibiotics despite their high similarities (Clarke et al., 2005), or the identification of 16 staphylococcal species (Rebrošová et al., 2017). One strategy to avoid the spectral complexity of biological systems consists in enhancing the contribution of the target molecules (i.e., microbial metabolite) employing a laser line with an excitation frequency that is in resonance with an absorption band of the analyte as in SERRS (Bodelón et al., 2016, 2017). On the downside, SERRS analysis of resonant-active metabolites may preclude the detection of non-resonant molecules also present in the sample. The Raman cross-section of the analyte is an important feature to be considered. In general, heterocyclic molecules containing aromatic rings are characterized by high Raman scattering activities. In addition, π-conjugated biomolecules tend to have strong Raman scattering cross-sections, owing to their distributed electron clouds that can be easily polarized in the presence of an electric field (Laing et al., 2017). Remarkably, many antibiotics, chromophore-containing molecules, and other metabolites that may be regulated by QS are characterized by having potential Raman-active features. Obviously, the *a priori* knowledge of the spectral fingerprint of the target biomolecule is essential in SERS studies. However, when aiming at the identification/detection of a target biomolecule produced by microbial cells (i.e., pyocyanin), the origin of the SERS signal must be unequivocally ascertained by the use of mutant strains deficient in the production of the biological compound.

The main challenge to obtain reliable SERS measurements is represented by the performance of the plasmonic substrates. For analytical applications, the preparation of these structures has to be straightforward and reproducible, while at the same time the signal enhancement has to be homogenous. SERS may suffer from issues related to substrate degradation that results in signal decrease over time. For instance, *in situ* measurements of living organisms (i.e., biofims) by SERS requires plasmonic devices that will have to withstand high ionic strength conditions, which may produce detrimental outcomes. Other significant issues are related with homogeneity and reproducibility of the SERS signal within the plasmonic substrate. They consist in the difficulty to generate uniform distributed enhancement factors, occurring only at localized positions (i.e., hot-spots) and the polydispersity of SERS-active colloidal clusters, which may hamper quantitative analysis. Although SERS often requires optimization of the plasmonic sensing system for each target analyte, new approaches have been developed to overcome these limitations and produce SERS-active substrates with high sensitivity, stability and reproducibility. The engineering of hot-spots and plasmonic supercrystals may circumvent some of the aforementioned problems (Shiohara et al., 2014; Scarabelli et al., 2016). Hamon and Liz-Marzán recently reviewed the most important parameters that should be considered in order to address the major issues associated to SERS when using conventional colloidal chemical synthesis, namely reproducibility, simplicity, selectivity, high throughput and sensitivity (Hamon and Liz-Marzán, 2018). In this framework, Cardinal and collaborators have also recently suggested practical considerations to facilitate SERS spectra reproducibility across different laboratories (Cardinal et al., 2017). Although commercial SERS-active substrates are available (Mosier-Boss, 2017), they are in general prepared by physical deposition methods, and not from wet chemistry approaches, which would provide well-defined surfaces, tailored nanoscale features, thereby improving the reproducibility of the measurements (Hamon and Liz-Marzán, 2018). Current high-performance SERS substrates are synthesized in academic laboratories under highly optimized conditions, thus large-scale production of such sophisticated devices with high reproducibility can be challenging. In general terms, in order to broaden the use of SERS and translate its application into the real world (e.g., clinical settings), it would be necessary the standardization and automatization of the procedures for the synthesis and functionalization of plasmonic transducers. Continuous development and improvement in Raman instrumentation, analytical workflow, and software are also crucial.

## Conclusions and outlook

Herein we have presented some recent applications of SERS spectroscopy for assessing QS in *P. aeruginosa*, such as detection and quantification of QS signaling molecules, chemical analysis of biofilm formation, *in situ* imaging of QS-regulated metabolites, as well as the use of SERS as a potential tool for screening protein-ligand interactions. In spite some limitations and challenges that still must be overcome, this highly versatile technique offers great potential for the study of extracellular metabolites and other secreted factors produced by microbial populations. The ability to visualize these chemical substances is fundamental to provide new knowledge into their function, as well as the spatiotemporal dependencies required for the chemical interactions shaping microbial communities. In this context, revealing the extensive intercellular signaling potential of bacteria, and other microbial species, can prove breeding ground to yield valuable ecological insight and drug prospecting.

With the prevalence of multidrug resistant bacteria, new antibiotics and therapeutic approaches are urgently needed. Specifically, the capacity of SERS to non-invasively study microbial populations may open new avenues for understanding QS and for the development of new therapies targeting this form of bacterial communication. Microorganisms represent a depository for natural-product discovery, many of which have been shown to be under QS regulation. Holistic approaches for the cultivation of microorganisms are being actively investigated in the search for new antimicrobials and for the development of bioactive substances that can function as antitumor agents, immunosuppressants or cholesterol lowering agents to name just a few (Nai and Meyer, 2017). Importantly, many of these bioactive molecules, some of which contain aromatic compounds, or have been shown to be pigments and chromophores, are amenable to SERS detection. In this context, recent technological advances in microscale cultivation devices provide a window of opportunity to transform the study of microbial communication, as well as to facilitate the discovery of new bioactive substances (Srinivasan et al., 2015). The success of these methodologies for studying bacterial populations would benefit from the adaptation of analytical tools able to detect trace amounts of the secreted metabolites, as well as their *in situ* characterization, applications for which SERS excels. In this framework, SERS is already being implemented in lab-on-a-chip and nano/microfluidics technologies for sensing in nanoliter volumes (Jahn et al., 2017).

The advancements in nanotechnology and photonics have dramatically incremented the capabilities of SERS, by which this analytical tool is increasingly being adopted in microbiology studies for very diverse applications, including the sensitive detection of pathogenic bacteria (Liu et al., 2017), and culture-free investigation of bacterial cells (Lorenz et al., 2017). As shown herein, SERS spectroscopy has great potential to be incorporated to the set of label-free methodologies already in use for revealing the “hidden” chemistry of microbes such as imaging mass spectrometry and conventional Raman spectroscopy. The advent of portable Raman spectrometer systems makes real-time, on-site, SERS monitoring of analytes an exciting possibility. Additionally, the extraordinary capability of SERS for the detection of analytes in trace amounts could prove to be a great asset for the early detection and diagnosis of infectious diseases.

## Author contributions

GB and IP-S conceived and wrote most of the work. VM-G and JP-J participated in the writing of the Raman scattering and SERS spectroscopy section. All listed authors actively contributed in the revision of the manuscript.

### Conflict of interest statement

The authors declare that the research was conducted in the absence of any commercial or financial relationships that could be construed as a potential conflict of interest.
